# 

**Published:** 1839-04

**Authors:** 


### BOOKS RECEIVED FOR REVIEW.

ENGLISH.

1. On Granular Degeneration of the Kidneys, and its connexion with Dropsy, Inflammations, and other Diseases. By Robert Christison, M.D., F.R.S.E., Professor of Materia Medica, &c. in the University of Edinburgh.—Edinburgh, 1839. 8vo. pp. 288. 8s.

2. A Letter to Dr. Chambers, F.R.S., &c. on several important points relating to the Nature and proper Treatment of Gout. By Sir Charles Scudamore, M.D., F.R.S., &c. —London, 1839. 8vo. pp. 59. 2s. 6d.

3. The Discovery of the Vital Principle, or Physiology of Man.—London, 1838. 8vo. pp. 566.

4. Elements of the Pathology of the Human Mind. By Thomas Mayo, M.D., F.R.S., &c.—London, 1838. 12mo. pp. 182.

5. Essays on the most important Diseases of Women. By Robert Ferguson, M.D., Professor of Obstetric Medicine, King's Coll. Lond.—London, 1839. 8vo. pp. 299.

6. On the method of Induction, and its results on Medical Science. An Introductory Lecture at King's Coll. Lond. Oct. 1, 1836.—London, 1839. 8vo. pp. 36.

7. Elements of Physiology. By J. Müller. Translated by W. Baly, M.D. Part IV.—London, 1838. 8vo. 4s.

8. An Exposition of Quackery and Imposture in Medicine; being a Popular Treatise on Medical Philosophy. By the Author of "The Philosophy of Living;" with Notes by W. Wright, Surgeon-Aurist. London, 1839. 8vo. pp. 259.

9. An Account of some Experiments on the Blood, in connexion with the Theory of Respiration. By John Davy, M.D., F.R.S. (From the Phil. Trans. Part II. for 1838.) —London, 1838. 4to. pp. 20.

10. Illustrations of Osteology. By Theodore S. G. Boisragon, M.D. Large Folio, 3 plates.—London, 1839. 6s.

11. Lexicon Medicum; or Medical Dictionary: containing an explanation of the terms in Anatomy, &c. &c. By the late Robert Hooper, M.D. Seventh Edition, revised, corrected, and enlarged, by Klein Grant, M.D., &c. — London, 1839. 8vo. pp. 1408. 30s.

12. Thoughts and Observations upon Pauperism, Poor Laws, Emigration, and the Prevention of Crime. By W. Fergusson, M.D., F.R.S.E.—London, 1839. 8vo. pp. 23. 1s.

13. Researches in Embryology. First Series. By Martin Barry, M.D., F.R.S.E. (From the Phil. Trans. for 1838.)—London, 1839. 4to. pp. 40. 8 plates.

14. Observations on the Comparative State of Medicine in France, England, and Germany, during a journey in those countries in 1835. By Dr. Adolph Muehry. Translated from the German, by E. G. Davis, M.D. (From Dunglison's American Library.) Philadelph. 1838. 8vo. pp. 126.

15. A Discourse commemorative of Philip Syng Physick, M.D. By Chas. Caldwell, M.D., Louisville (United St.) 1838. 8vo. pp. 41.

16. On long-continued contraction of the Lower Extremities, from an affection of the Spine. By R. A. Stafford, Esq. (From Med. Chir. Trans.)—London, 1839. 8vo. pp. 26.

17. An Experimental Inquiry concerning the presence of Alcohol in the Ventricles of the Brain, after poisoning by that liquid; together with Experiments illustrative of the physiological action of alcohol. By John Percy, M.D.—London, 1839. 8vo. pp. 112.

18. Vital Statistics of Glasgow. Statistics of Fever and Smallpox. By Robert Cowan, M.D.—Glasgow, 1838. 8vo. pp. 54. 1s.

19. Elements of Chemistry. By the late Dr. Turner. Sixth Ed. enlarged and revised. Part III. No. 1.—Lond. 1839. 8vo. 3s. 6d.

20. Illustrations of the Botany and other branches of the Natural History of the Himalayan Mountains, and of the Flora of Cashmere. By J. F. Royle, M.D., V.P.R.S., Professor of Materia Medica, King's College. Part X.—London, 1839. Folio. 20s.

21. Medical Portrait Gallery. By T. J. Pettigrew, F.R.S. No. 13. Containing Memoirs and Portraits of Boerhaave and Mr. Travers.—London, March, 1839. 3s.

22. On the Physiology or Mechanism of Blushing; illustrative of the influence of mental emotion on the Capillary Circulation; with a general view of the sympathies and the organic relations of those structures with which they seem to be connected. By T. H. Burgess, M.D.—London, 1839. 8vo. pp. 202.

23. Woman physiologically considered as to Mind, Morals, Marriage, Matrimonial Slavery, Infidelity, and Divorce. By Alex. Walker.—London, 1839. 8vo. pp. 464.

24. Vegetable Organography; or, an analytical description of the Organs of Plants. By M. A. P. De Candolle, &c. Translated by Boughton Kingdon. With plates. To be completed in 16 parts. Parts I., II., 2s. 6d. each.—London, 1839. 8vo.

25. Illustrations of Cutaneous Disease. A series of delineations of the affections of the Skin, in their more interesting and frequent forms; with a practical summary of their Symptoms, Diagnosis, and Treatment, including appropriate Formulæ. By R. Willis, M.D., &c. Fasciculi I. II. III., each containing 4 plates.—Lon., 1839. Fol. 5s.

FOREIGN.

[Want of room obliges us to postpone to our next Number, the List of Foreign Books received for review during the last quarter.]

### INDEX TO VOL. VII.

OF THE

### BRITISH AND FOREIGN MEDICAL REVIEW.

| | PAGE |
|---|---|
| ABORTION, Dr. Jörg on . . | 135 |
| Acephalocyst, Dr. Bright on . | 462 |
| Aitken, Dr. his physiology . | 214 |
| Almanac, medical, Mr. Farr's . | 294 |
| Allen, Dr. on the insane . . | 1 |
| Ammonia as a counter-irritant . | 63 |
| Amenorrhœa, singular remedy in . | 234 |
| Aneurisms of the heart . . | 149 |
| Aneurism, popliteal, case of . | 153 |
| ventral, enormous . | 154 |
| Animal Magnetism, treatises on . | 301 |
| history of . | 303 |
| in France . | 309 |
| in Germany . | 315 |
| Gmelin's views of | 318 |
| acts at a distance | 328 |
| morality of . | 335 |
| in England . | 347 |
| Anæsthesia, case of . . | 544 |
| Aorta, wound of, cured . . | 251 |
| aneurism of, signs of . | 366 |
| Apoplexy of infants . . | 92 |
| Areola, mammary, veins of . | 240 |
| Arsenic, its use in uterine diseases | 151 |
| case of recovery from . | 563 |
| Artery basilar, structure of . | 574 |
| Ashwell, Dr. on uterine hemorrhage | 450 |
| his obstetric cases . | 451 |
| Association, Provincial, on influenza | 95 |
| Auscultation in infantile pneumonia | 79 |
| obstetrical . . | 230 |
| cerebral . . | 268 |
| Autoplasty, or plastic surgery . | 386 |
| Baron, Dr. on smallpox and cowpox | 295 |
| Bardsley, Dr. on homœopathy . | 540 |
| Beaumont, Mr. on fistula . | 142 |
| Bigsby, Dr. on colica pictonum . | 284 |
| Bilious disorders, dialogue on . | 476 |
| Birmingham, Medical School of . | 520 |
| Blakiston, Dr. on influenza . | 95 |
| Blandin, M. on erysipelas . | 252 |
| plastic surgery . | 386 |
| Bischoff, Prof. on transfusion of blood | 548 |
| Blepharoplasty . . | 404 |
| Blood, diabetic, analysis of . | 457 |
| on transfusion of . | 548 |
| and respiration, relations of | 279 |

| | PAGE |
|---|---|
| Bluff, Dr. obituary of . . | 298 |
| Boisragon, Dr. on the bones . | 537 |
| Bone, on the absorption of dead . | 140 |
| Bones, on the composition of . | 154 |
| pathology of . . | 566 |
| Books for review . | 299, 594 |
| Bostock, Dr. on the urine . | 142 |
| Bright, Dr. on abdominal tumours | 462 |
| Broussais, M. obituary of . | 297 |
| Bronchi, spike of oats in . | 251 |
| Bronchocele, encysted, case of . | 586 |
| Bruit de soufflet, on the . | 276 |
| Burchard, Dr. on tumours of the scalp | 74 |
| Burns, new mode of treating . | 287 |
| Burnett, Mr. on the Divine attributes | 227 |
| Calculi, on solution of . . | 166 |
| Calculous diseases in seamen . | 141 |
| Camphor, a panacea . . | 549 |
| Causes of disease, their importance | 120 |
| Cancer of the face . . | 144 |
| Capsules, supra-renal . . | 241 |
| Carpenter, Mr. his physiology . | 168 |
| Carswell, Dr. on inflammation . | 417 |
| Cartilage, pathology of . . | 535 |
| Casts, anatomical, new . . | 278 |
| Catamenia, source of . . | 233 |
| Cephalæmatoma . . | 86 |
| Cerebro-spinal phenomena . | 220 |
| Chambers, Dr. letter to . . | 534 |
| Chatham, museum of . . | 224 |
| Chest, mensuration and inspection of | 353 |
| normal and altered forms of . | 354 |
| Cheiloplasty . . | 407 |
| Chemical tests, treatise on . | 533 |
| Children, mortality of . . | 293 |
| cerebral tubercles in . | 576 |
| Child, enormous weight of . | 586 |
| Cholera, report on . . | 149 |
| Churchill, Dr. on prolapsus of the cord | 291 |
| Chromium, experiments with . | 564 |
| Cicatrix vaccine, its value . | 203 |
| Circulation, physiology of . | 179 |
| Clavicle, extirpation of . . | 147 |
| Clarke's, Mr. singular case of poisoning | 575 |
| Clendenning, Dr. on the heart . | 142 |
| Cock, Mr. on deafness . . | 451 |
| Colica pictonum, use of oil in . | 284 |

PAGE

Constipation, mode of treating . 478

Consumption, Dr. Furnivall on . 536

Cooper, S. his surgical dictionary . 526

Cooper, Sir A. on varicocele . 444

Mr. B. on fracture . 450

on the muscles of the eye 457

Coroner, his office and duties . 589

Corrigan, Dr. on bruit de soufflet . 276

Counter-irritation, treatises on . 56

Counter-irritant lotions . . 292

Cowpox, history of, in Wirtemberg 186

complications of . 192-200

modifications of . 192

degree of protection from 206

identity with smallpox . 295

Cows, variety of pocks in . 190

Crowther, Dr. on madhouses . 2

Cross-breeding, principles of . 379

Cruveilhier on ulcer of stomach . 241

Cupping a counter-irritant . 69

Cutaneous diseases of infants . 93

Davy, Dr. on the blood . . 279

basilar artery . 574

organic disease . 575

Deafness, congenital, pathology of 451

Deglutition of a coin . . 255

Delirium tremens, treatment of . 268

Development of organs, laws of . 176

Diabetes mellitus . . 164

insipidus . . 160

Dieffenbach's operations on the face 250

Dr. on plastic surgery . 386

on wry-neck . 560

Digestion of the stomach . 524

Dumenil, Dr. on chemical tests . 533

Dunglison, Dr. his physiology . 229

Elephantiasis, tremendous case of 578

Ellis, Sir W. on insanity . 1

Emmenological questions . 233

Emphysema, pulmonary, signs of . 365

on curing of . 516

Episioraphy, Dr. Fricke on . 562

Epps, Dr. on counter-irritation . 56

Erysipelas, theory and treatment of 252

Esquirol, M. on insanity . 1

Eye, singular adaptation of . 228

on the muscles of . . 457

Eyelids, restoration of . . 404

Face, malignant diseases of . 143

removal of bones of . 250

Fallopian tube, pregnancy of . 562

Farr, Mr. on lunatic asylums . 2

his medical almanac . 294

Females, responsibility of, in pregnancy 129

Ferguson, Dr. on puerperal fever . 482

Ferrarese, Dr. on insanity . 1

Fever, miliary, epidemic . 244

puerperal, Dr. Ferguson on 482

varieties of . 483

symptoms of . 484

anatomy of . 487

nature of . 490

PAGE
Fever, puerperal, treatment of . 495
Fistula lachrymalis . . 410
Finger, swelling of, from rings . 515
Flora Medica, Dr. Lindley's . 531
Flourens, M. on the skin . 239
Fluids, vitiated, in puerperal fever 490
Fœtus, its value . . 133
Food, Mr. Grisenthwaite on . 538
Fracture bandage, immoveable . 254
Fractures, reparation of . . 450
of the ribs . . 554
new splints for . 583
Fricke, Dr. on Episioraphy . 562
Frog, on the nerves of . 237, 541
Furnivall, Dr. on consumption . 536
Gangrene, infantile, account of . 470
Gardner, Mr. on kephalosis . 222
Generation, physiology of . 184
Gestation, period of . . 136
Gluge, Dr. on influenza . . 95
Gondret, M. on counter-irritation . 56
Goodsir, Mr. on the teeth . 571
Gout, Sir C. Scudamore on . 534
Gorham, Mr. on intussusception . 453
Granville, Dr. on counter-irritation 56
his lotions . . 292
Grant, Dr. K. his Hooper's dictionary 526
Greco, Dr. on insanity . . 1
Greenhow, Dr. on burns . . 287
Green, Dr. on infantile mortality . 293
Greene, Dr. on nervous tremor . 583
on cerebral tubercles . 576
Gas in the vascular system . 249
Gulliver, Mr. on absorption of bone 140
Grisenthwaite, Mr. on food . 538
Guy, Dr. on the pulse . . 458
Guy's Hospital Reports . 443
Hawkins, Mr. on diseases of the face 143
Hayward, Dr. his physiology . 214
Heat, as a counter-irritant . 62
animal, Hunter's view of . 524
Heart, concentric hypertrophy of . 153
weight of . . 142
Hedenus, Dr. obituary of . 298
Heim, Dr. on vaccination . 186
Hemorrhage, uterine, from tumours 451
Henry, Dr. on bilious complaints . 476
Hernia, causes of strangulation in . 456
mesenteric, case of . 290
case of . . 584
Hohnbaum, Dr. on ventral pulsation 511
Hooker, Dr. on respiration . 263
Hooper's medical dictionary . 526
Hooping-cough, Dr. Lombard on . 283
Dr. Roe on . 210
Hunter, John, on inflammation . 417
his style . . 419
on the animal economy 523
Hunt, Mr. on the use of arsenic . 151
Hutchinson, Mr. on calculus in seamen 141
on revaccination . 581
Hybernation, Hunter on . . 524

| | PAGE |
|---|---|
| Hydrophobia, treatment of | 270 |
| Hydrocyanic acid, its properties | 572 |
| Hysteria, Mr. Laycock on | 282 |
| Infanticide, suspected | 257 |
| Infants, evil of feeding by hand | 569 |
| Rau on the mortality of | 591 |
| diseases of new-born | 74 |
| mode of quieting | 77 |
| on the gangrene of | 470 |
| Inflammation, John Hunter, &c. on | 417 |
| Dr. Macartney on | 221 |
| treatment of | 442 |
| of vermiform process | 551 |
| Influenza, history of | 95 |
| Insane, on the classification of | 1 |
| Insanity, treatises on | 1 |
| cases of | 9 |
| treatment of | 34 |
| Ischuria renalis | 160 |
| Intermarriage, Mr. Walker on | 370 |
| Intussusception in infants | 453 |
| Jörg on the responsibility of women | 129 |
| Jurisprudence, medical, Most on | 530 |
| Kennion, Dr. on the optic nerve | 282 |
| Kephalosis, Mr. Gardner on | 222 |
| Key, Mr. on spinal disease | 446 |
| King, Mr. on strangulated hernia | 456 |
| Kinnis, Dr. on vaccination | 527 |
| King, Dr. on amenorrhœa | 233 |
| Lauterbourg, a magnetiser in England | 312 |
| Law on the administration of mercury | 581 |
| Lead, action of water on | 448 |
| Leighton on medical science | 525 |
| Life, nature of | 171 |
| Lindley, Dr. his Flora Medica | 531 |
| Lips, restoration of | 407 |
| Liquor amnii, analysis of | 457 |
| Liston, Mr. on plastic surgery | 386 |
| his surgery | 235 |
| Lithotomy, statistics of, in Naples | 262 |
| new operation for | 505 |
| Lithotrity, Mr. Ferguson on | 286 |
| Liver, enlarged, new signs of | 146 |
| Lizars, Mr. his surgery | 231 |
| Lombard, Dr. on emphysema | 516 |
| hooping-cough | 283 |
| Lonsdale, Dr. on hydrocyanic acid | 572 |
| Lord, Mr. his physiology | 214 |
| Lower jaw, excision of | 152 |
| Lunatic Asylum, reports of | 1 |
| Lunatics, mortality of | 22 |
| Macartney, Dr. on inflammation | 417 |
| Madhouses, Dr. Crowther on | 2 |
| Magnetism, animal, history of | 301 |
| Magnesia, effervescent | 570 |
| Malcolmson, Mr. on diseased liver | 146 |
| Malgaigne, M. on fractured ribs | 554 |
| Marriage and insanity | 27 |
| Medicine a composite science | 115 |
| its difficulties | 116 |
| Medulla spinalis, Mr. Hilton on | 277 |
| Medical jurisprudence, cases in | 454 |

| | PAGE |
|---|---|
| Meloplasty | 408 |
| Mercury, external use of, in smallpox | 552 |
| in small doses, effect of | 581 |
| Mesmer and Mesmerism | 305 |
| Meteorological observations | 294 |
| Milk-thrush of infants | 82 |
| Modelling process, Macartney on | 426-430 |
| Mortality of New York | 272 |
| Most, Dr. on medical jurisprudence | 530 |
| Mouth, restoration of | 408 |
| Muscæ volitantes, cause of | 280 |
| Nerves of the frog | 237 |
| structure of | 500 |
| Nervous affections of young women | 146 |
| Neuralgia, Dr. Rowland on | 529 |
| Nose, restoration of | 390-400 |
| Obstetrical enormity | 570 |
| Optic nerves, origin of | 282 |
| Ophthalmia, purulent, treatment of | 155 |
| Dr. Slade on | 217 |
| Organic disease, prevalence of | 575 |
| Ovaries, on tumours of | 464 |
| Owen, Mr. his notes on Hunter | 523 |
| Oxalic acid, poisoning by | 454 |
| Palate, restoration of | 411 |
| Parents and offspring | 375 |
| Paralysis, Mr. Key on | 446 |
| partial, cases of | 216 |
| facial, cases of | 247 |
| Parker, Mr. on the stomach | 115 |
| Peritonitis in the pelvis | 284 |
| Pericarditic effusions, signs of | 368 |
| Peritoneum in perforation of intestines | 248 |
| Pettigrew, Mr. his portrait gallery | 225 |
| Physiology, popular, works on | 214 |
| Dr. Dunglison's | 225 |
| Mr. Carpenter's | 168 |
| Placenta, absorption of | 255 |
| Pleuritic effusion, signs of | 366 |
| Pneumonia of infants | 77 |
| Pneumothorax, cause of sounds in | 569 |
| Poisoning by the rattlesnake | 578 |
| by arsenic, cured | 563 |
| Polypi, new mode of tying | 253 |
| Portrait gallery, medical | 225 |
| Pregnancy, extra-uterine | 257 |
| of the Fallopian tube | 562 |
| Prepuce, restoration of | 412 |
| Prichard, Dr. on insanity | 1 |
| Printing on gold, poisonous | 291 |
| Prophetess of Prevorst | 339 |
| Prostitution in London | 539 |
| Puerperal fever, Dr. Ferguson on | 482 |
| Pulse, effect of posture on | 458 |
| of the fœtus | 230 |
| its frequency in infants | 76 |
| Pulsation, epigastric | 511 |
| Purgatives, condemnation of | 477 |
| causes of their popularity | 478 |
| Pus, deposition of, in joints | 149 |
| Pustula maligna | 550 |
| Quinsy, scarification in | 550 |

| | PAGE |
|---|---|
| Rainy, Dr. on urea in the blood . | 586 |
| Rasori, Dr. on inflammation . | 417 |
| Raspail, M. on camphor . | 549 |
| Rau, Dr. on the mortality of infants | 591 |
| Rees, Dr. on bone . . | 154 |
| Remak, Dr. on the structure of nerves | 500 |
| Reparation of parts, not inflammatory | 426 |
| Reproduction, physiology of . | 188 |
| Resemblance of children to parents | 275 |
| Respiration and circulation, relations of | 263 |
| Reunion of divided parts . | 429 |
| Revaccination at the Foundling Hosp. | 581 |
| in Wirtemberg . | 186-203 |
| in the Prussian army | 567 |
| modifications in . | 208 |
| discussion on . | 568 |
| Rhinoplasty . . | 400 |
| Ribs, on fracture of . . | 554 |
| Richter, Dr. on infantile gangrene | 470 |
| Roberts, his case of injury of the brain | 585 |
| Roe, Dr. on hooping-cough . | 210 |
| Romberg, Dr. on the fifth nerve . | 544 |
| Rowland, Dr. on neuralgia . | 529 |
| Ryan, Dr. on prostitution . | 539 |
| Saliva, analysis of . . | 236 |
| Scalp, bloody tumour of . | 74 |
| Scarification in quinsy . . | 550 |
| Schumer, Dr. on cartilages . | 535 |
| Scudamore, Sir C. on gout . | 534 |
| Sebastian, Prof. on tubercle . | 553 |
| Sediments, urinary . . | 160 |
| Selwyn, Dr. on bronchocele . | 586 |
| Sensation, loss of, in the fifth nerve | 544 |
| Simpson, Dr. on fœtal peritonitis . | 284 |
| Skrimshire, Dr. his Medical Guide | 513 |
| Skin, on the structure of . | 239 |
| Slade, Dr. on ophthalmia . | 217 |
| Smallpox, Mr. Eagle on . . | 278 |
| in Wirtemberg . | 186 |
| local use of mercury in . | 552 |
| Smee, Mr. his new splints . | 583 |
| Somnambulism, magnetic . | 331 |
| Spinal marrow, structure of . | 502 |
| cord, collection of serum in . | 248 |
| marrow, structure of . | 541 |
| Spirits and ghosts, mesmeric . | 342 |
| Spleen, Dr. Bright on diseases of . | 464 |
| Staphyloplasty . . | 411 |
| Stevens, Dr. on lithotomy . | 505 |
| Stimuli, vital . . | 175 |
| Stomach, on ulcer of . . | 241 |
| morbid states of . | 115 |
| Stomatoplasty . . | 408 |
| Streeten, Dr. on influenza . | 95 |
| Suicide from strangulation . | 261 |
| Surgery, plastic, works on . | 386 |
| Sweich, Dr. on influenza . | 95 |
| Sympathetic nerve, structure of . | 541 |
| Syphilis, hydriodate of potass in . | 245 |
| Taliacotius, his plastic operations . | 388 |
| Taylor, Mr. on the action of lead . | 448 |
| his cases in med. surgery | 454 |
| Teeth, on the origin of . . | 571 |
| structure of . . | 276 |
| Temperature, effect of, on wounds . | 251 |
| of uterus and vagina . | 549 |
| Temple, magnetic . . | 331 |
| Tendons, cicatrization of . | 240 |
| Tetanus successfully treated . | 559 |
| Thomas, Mr. his address . | 520 |
| Thrush of infants . . | 82 |
| Thurnam, Mr. on aneurism . | 149 |
| Trachea, fistula of . . | 411 |
| Thyroid gland, dropsy of . | 586 |
| Transactions, medico-chirurgical . | 140 |
| Travers, Mr. removal of clavicle by | 147 |
| Tubercles, origin of . . | 553 |
| Tumour, bloody, of the scalp . | 86 |
| Tumours, abdominal . . | 462 |
| Tyrrell, Mr. on purulent ophthalmia | 155 |
| Umbilical cord, prolapsus of . | 291 |
| Urea in the blood, in cholera . | 586 |
| Urethra, restoration of . . | 413 |
| mode of dilating . | 290 |
| Urine, Dr. Bostock on . . | 142 |
| Urinary diseases . . | 155 |
| Uterus, temperature of . . | 549 |
| Vaccination in Ceylon . . | 527 |
| in Wirtemberg . | 186 |
| proper age for . | 196 |
| mode of performing . | 196 |
| Vagina, temperature of . . | 549 |
| Valleix, M. on diseases of infants . | 74 |
| Varicocele, Sir A. Cooper on . | 444 |
| Varicose veins, treatment of . | 254 |
| Vermiform process, inflammation of | 551 |
| Vesico-vaginal fistula . | 289, 415 |
| Viscera, transposition of . . | 249 |
| Vision, binocular . . | 279 |
| Wallace, Dr. on muscæ volitantes . | 280 |
| Water-canker, Richter on . | 471 |
| Waugh on cerebro-spinal phenomena | 228 |
| Willis, Dr. on urinary diseases . | 155 |
| Wilson, Mr. his case of hernia . | 584 |
| Dr. on nervous affections . | 146 |
| Woillez, M. on examining the chest | 353 |
| Women, Ferguson on the diseases of | 482 |
| Wound of the head, fatal from neglect | 259 |
| Wry-neck, cure of . . | 560 |
| Zeis, Dr. on plastic surgery . | 386 |
| Zinc, medicinal effects of . . | 567 |

END OF VOL. VII.

PRINTED BY C. ADLARD, BARTHOLOMEW CLOSE.

